# Validity and reliability of a structured interview for early detection and risk assessment of parenting and developmental problems in young children: a cross-sectional study

**DOI:** 10.1186/1471-2431-12-71

**Published:** 2012-06-14

**Authors:** Henk F van Stel, Ingrid I E Staal, Jo M A Hermanns, Augustinus J P Schrijvers

**Affiliations:** 1 Julius Center for Health Sciences and Primary Care, University Medical Center Utrecht, Heidelberglaan 100, 3584 CX, Utrecht, the Netherlands; 2 Department of Preventive Child Health Care, Municipal Health Service Zeeland, Goes, the Netherlands; 3 Faculty of Social and Behavioural Sciences, University of Amsterdam, Amsterdam, the Netherlands

## Abstract

**Background:**

Preventive child health care is well suited for the early detection of parenting and developmental problems. However, as far as the younger age group is concerned, there are no validated early detection instruments which cover both the child and its environment. Therefore, we have developed a broad-scope structured interview which assesses parents’ concerns and their need for support, using both the parental perspective and the experience of the child health care nurse: the Structured Problem Analysis of Raising Kids (SPARK). This study reports the psychometric characteristics of the SPARK.

**Method:**

A cross-sectional study of 2012 18-month-old children, living in Zeeland, a province of the Netherlands. Inter-rater reliability was assessed in 67 children. Convergent validity was assessed by comparing SPARK-domains with domains in self-report questionnaires on child development and parenting stress. Discriminative validity was assessed by comparing different outcomes of the SPARK between groups with different levels of socio-economic status and by performing an extreme-groups comparison. The user experience of both parents and nurses was assessed with the aid of an online survey.

**Results:**

The response rate was 92.1% for the SPARK. Self-report questionnaires were returned in the case of 66.9% of the remaining 1721 children. There was selective non-reporting: 33.1% of the questionnaires were not returned, covering 65.2% of the children with a high-risk label according to the SPARK (p < 0.001). Inter-rater reliability was good to excellent with intraclass correlations between 0.85 and 1.0 for physical topics; between 0.61 and 0.8 for social-emotional topics and 0.92 for the overall risk assessment. Convergent validity was unexpectedly low (all correlations ≤0.3) although the pattern was as expected. Discriminative validity was good. Users were satisfied with the SPARK and identified some topics for improvement.

**Conclusion:**

The SPARK discriminates between children with a high, increased and low risk of parenting and developmental problems. It does so in a reliable way, but more research is needed on aspects of validity and in other populations.

## Background

Early detection of parenting problems and problems in the psychosocial development of young children is important [1-7], as interventions are supposedly more effective when they are carried out earlier [6-12]. Evidence shows that this early detection is preferably done by using a validated instrument [7,13,14].

In the Netherlands, the law requires preventive child health care (CHC) to detect parenting and developmental problems at an early stage. However, as the younger age group is concerned, there are no validated early detection instruments which cover both the child and its environment. Therefore, we have developed the Structured Problem Analysis of Raising Kids (SPARK)[15]. The SPARK is a structured interview for early detection and risk assessment of parenting and developmental problems in young children. This instrument combines the perspectives of the parent(s) and the professional. The SPARK asks parents to voice any concerns and problems on a broad range of topics, and then to indicate the need for support perceived by both parent and CHC-professional, followed by a joint decision on subsequent care. It finishes with a structured overall risk assessment for parenting and developmental problems by the professional.

The development study of 1140 children shows that the SPARK is discriminative and practicable [15]. Before the SPARK can be further implemented in clinical practice, further study is needed on the psychometric characteristics of this instrument. As no criterion instrument (‘gold standard’) exists for early detection of parenting and developmental problems, criterion validity cannot be assessed. Therefore, we have assessed the SPARK on interrater reliability, convergent validity, discriminative validity, and the user experience of both parents and CHC-professionals.

## Methods

### Study design

We performed a cross-sectional study on all children living in the province of Zeeland and born between January 15 and July 31 2006, a total of 2012 children. Once a month, all children who would reach the age of 18 months the following month were identified in the municipal population registry. This has the goal that all eligible children could be contacted. The CHC nurse contacted parents for the regular check-up at the age of 18 months, which consisted of a home visit by the CHC nurse or a visit to the well-baby clinic by parent(s) and child, and included an information letter on the aim of the visit and the primary study (assessing the value of a structured interview during home visits and visits to the well-baby clinic). The visit started with the structured interview (SPARK), with the primary goal of deciding together with the parent(s) which type of (health) care was needed by child and parent(s). The interview was followed by a request (verbal + written) for informed consent to use the information recorded in the SPARK for scientific research. The order of the steps was chosen on purpose, as it would be complicated to discuss parenting problems and care needed after informed consent was denied. The CHC nurses were not aware of the study goals of the validation study to prevent bias. The study protocol was approved by the Medical Ethical Review Committee of the University Medical Center Utrecht.

Reliability of the SPARK was assessed by the inter-rater agreement. In a random sample of 67 children a second CHC nurse was also present. Her function was to listen to the interview, without interfering, and to fill in the SPARK-form independently from the interviewing CHC nurse. Convergent validity was assessed by comparing SPARK-domains with domains in self-report questionnaires on child development and parenting stress which cover concepts also addressed in the SPARK. Parents who gave informed consent were requested to complete a set of questionnaires (described below). Discriminative validity was assessed by comparing different outcomes of the SPARK between groups with different levels of socio-economic status (SES) and by performing an extreme-groups comparison. We hypothesized that children from families with lower SES would report more problems and need for support, and that this group would include more children with a high and increased risk of parenting problems. The extreme-groups comparison was done by comparing the mean levels of concern and perceived need for support and the risk assessment between *a)* all children with a confirmed report to the child protective services between birth and the age of 18 months (n = 21), and *b)* the ‘everything OK’ group: a group of children with normal scores on all self-report questionnaires and no known risk factors (which include large family (≥ four children), single parent, young parent (<20 years at birth of child), very low educational background of parents, parents not speaking Dutch at home, unemployed or unemployable parents)[16,17]. As the latter group was very large (n = 912), we took a random sample from this group of three times the number of the reported group. Again, children with a confirmed report were expected to show more problems and a higher risk.

### Instruments

The way the SPARK was conceived has been described in detail in a previous study [15]. The SPARK consists of 16 topics in the following order: infancy review (reviewing past issues and discussing any problems arising from the infant period that are still relevant); somatic health; motor development; language, speech and thought development; language use of parents (second language, mother tongue); emotional development; contact between the child and others (both children and adults); child behavior; parenting approach; developmental stimulation and early/preschool education; how the child spends its time; living environment in and outside the home; social contacts and informal support; day-care for the child; concerns communicated by others; family issues; and lastly a question about whether any topic has been forgotten or needs further attention. The SPARK uses a 3-step model: Step 1: detection of problems and concerns; Step 2: clarifying the characteristics and seriousness of problems and concerns in dialogue with the parents; Step 3: analysis and a decision on what to do next. For each topic, the CHC nurse starts with a short description of the topic with examples, and asks the parents if they have experienced any concerns, questions or problems in the last six months (Step 1). Parents are requested to assess the seriousness of these concerns on a five-point Likert scale presented on a printed card, ranging from “no concern at all” to “very concerned”. If concerns are cited, respondents are asked to elaborate on the exact nature of concerns, questions or problems, and whether or not professional and/or informal help – if offered – has been sufficient. Each topic ends with the parents assessing their current perceived need for support, on a six-point Likert scale: 1) no help needed; 2) information wanted; 3) personal advice; 4) counselling; 5) intensive help; 6) immediate intervention required. The CHC professional then makes the same assessment (Step 2). The information of steps 1–2 is recorded on a one-page form with a matrix structure: the first column includes all topics, followed by columns for each separate question: concerns / used support / support helped / current perceived need for support by parents / perceived need for support by nurse. After all the topics have been covered, the CHC nurse discusses with the parents the amount and content of care needed in the following months (Step 3), and notes this together with a description of the concern or problem on the second page, on which the possibilities for further care have been preprinted. Having done this, the CHC nurse ends the visit and subsequently makes an overall risk assessment on the third page, assigning the child a low, increased or high risk for parenting and development problems. The CHC nurse bases this overall risk assessment on the information from the interview, and on an elaboration of factors that might positively or negatively influence this risk assessment. This structured elaboration includes the observation of several factors, preprinted on the third page: the interaction between parent(s) and child(ren); growth and development of the child; manifest problems (both in the child such as existing illness, and in the family such as major life events, history of psychiatric illness, financial problems etc.); and living environment (hygiene, housing, family composition).

The set of self-report questionnaires on child development and parenting stress included a pre-stamped envelope addressed to the research team. The set consisted of the following questionnaires: 1) Ages and Stages Questionnaire (ASQ) version 2, 18-month version [18,19]. The ASQ consists of 30 questions on 5 domains: communication, gross motor, fine motor, problem solving and personal social. The ASQ has three answering options: ‘yes’, ‘sometimes’, ‘not yet’. Domains have a range of 0 to 60. 2) The Ages and Stages Questionnaire: Social Emotional (ASQ:SE, 18 month version) also has three answering options: ‘most of the time‘, ‘sometimes’ and ‘rarely or never’. Parents are asked to tick off a checkbox if the item in question is a concern [20]. The ASQ:SE has a scoring range of 0 to 255 in the 18-month version. 3) the short validated Dutch version of the Parenting Stress Index [21], called ‘Nijmeegse ouderlijke stress index – kort’ (NOSIK) [22]. The NOSIK consists of 25 items using a 6-point Likert scale ranging from ‘do not agree at all’ to ‘do completely agree’, with a scoring range of 25 to 150. 4) a partly validated questionnaire on psychological and pedagogic problems in young children which is frequently used in preventive CHC in the Netherlands: the ‘Kort Instrument voor de Psychologische en Pedagogische Probleem Inventarisatie’ (KIPPPI) [23]. This self-report questionnaire consists of 70 items grouped into a total score, and 19 yes/no items on life events.

The 18-month versions of both ASQ and ASQ:SE have been translated into the Dutch language using a double forward – once backward procedure. The (minor) differences have been resolved in cooperation with the developer of these questionnaires. Although these translations of the ASQ and ASQ:SE have not been validated, the ASQ and ASQ:SE have proven to be practicable and valid in other countries than the USA [24-26], including the Netherlands (48 month version [27]). Additionally, data have been gathered on demographic variables: age of father and mother at birth of first child, level of education of both parents, current working status of both parents, language spoken at home. Both the SPARK and the self-report questionnaires have been scanned using Teleform®. Socio-economic status (SES) has been assessed on neighborhood level: using the postal code for the house address of the child, each child has been assigned the SES-level of his or her neighborhood, using figures of Statistics Netherlands delivered by the Municipal Health Service of Zeeland. SES has been measured in 7 categories, from very low to very high. Most of the 155 postal code regions in Zeeland have a medium SES. For the extreme-groups comparison, we checked with the child protective services (Advice and Reporting Centres for Child Abuse and Neglect, and Youth Care Agency) which children in our sample had a confirmed report between birth and the age of 18 months.

For assessment of the user experience of both parents and CHC-professionals, we adapted a short questionnaire on CHC-nurses’ skills meant for increasing parents’ parenting competences [28]. During November 2007, parents and CHC-nurses were asked to complete this questionnaire online for each visit using the password-protected online survey tool NetQ (http://netq.nl).

### Statistical analysis

Reliability of the SPARK was assessed by the inter-rater agreement between the SPARK and a listen-only version as described above. We computed an intraclass correlation (ICC) using an 'observer nested within subject’ approach [29]. We only did this for the risk assessment and the need for support on the different topics as perceived by the CHC professional, as the answers given by the parents would be scored identically. Convergent validity of the SPARK was assessed by computing Spearman correlations between the care need expressed by parents and by CHC professionals on the 16 topics with domains in the self-report questionnaires. Using a multitrait-multimethod matrix [30] we expected higher correlations between related domains, such as motor development in the SPARK and gross motor in the ASQ; child behavior with ASQ:SE total score and NOSIK etc; and low correlations between differing domains such as physically oriented domains in the SPARK and parenting stress (the NOSIK score). Solely for the purpose of assessing discrimitative validity, we computed summary scores for concerns and perceived need for support by summing the scores for all topics and dividing by the number of topics. Thus, the scoring range of the summary scores was the same as with the original variables. Differences between postal code regions with different SES-levels on these summary scores for concerns or perceived need for support were tested using a Kruskal-Wallis test [31]. The extreme groups were compared using a Mann–Whitney U-test on concerns and perceived need for support, and a chi-square test on the risk assessment. Data-analysis was done using SPSS version 17. A p-value below 0.05 was considered significant.

## Results

During the study period 2012 eligible children were living in the province of Zeeland. No SPARK was received for 136 children (6.8%). For another 155 children, an incomplete SPARK was available. This group consisted of a) received with comment ‘no contact wanted by parents’ (n = 24); b) missing risk and/or consent data (n = 25); and c) no consent obtained after administration of the SPARK (n = 106)). Children for whom no SPARK was received, or an almost empty SPARK with the comment ‘no contact wanted by parents’, were counted as a non-response. From the remaining 1721 children, self-report questionnaires were returned for 1152 children (66.9%). Characteristics of the study population are described in Table 1. Administration of the SPARK took on average 29 minutes (standard deviation 11 min.). Table 2 shows scores per domain on parents’ concerns, needs assessment by parents and professional.

**Table 1:** Population characteristics (data only from the consent group, n = 1721)

Child characteristics:	
Male / female	53,5% / 46,5%
Place in family order:	
first child	41,7%
second child	36,7%
third child	13,8%
four or younger child	7,8% (max 12 children )
Family characteristics:	
2-parent household	92,5%
1-parent household	3,1%
shared household	2,7%
other (foster-family / adoption / divorcement /living with grandparents)	1,7%
Parent characteristics:	
age mother (mean in year, sd)	30,5 (sd 4,8)
mother aged < 20 yr at birth of this toddler	0,7% (n = 13)
age father (mean in year, sd)	33,4 (sd 5,8)
father aged < 20 yr at birth of this toddler	0,3% (n = 6)
Ethnicity: non-Dutch mother	8,7%
Ethnicity: non-Dutch father	7,8%
Language: non-Dutch used at home by mother	9,0%
Language: non-Dutch used at home by father	7,5%
Education:	
Low education	19,4% mother (including 2,3% very low) 21, 2% father (including 1, 9% very low)
Intermediate education	52,5% mother / 50,7% father
High education	28,1% mother / 28,1% father
Employment:	
Employed	72,7% mother / 92,9% father
Unemployed	1,1% mother / 0,9% father
Unemployable/unable to work	0,6% mother / 0,8% father
Stay-at-home mother/father	25,3% mother/ 0,8% father

**Table 2:** Scores per domain on parents’ concerns, needs assessment by parents and professional

**Domains:**	**Parents’ concerns:**	**Perceived need of support**				p-value*:
		Parents assessment*:		Professional assessment*:		
	**concerned/ very concerned**	**information wanted/ personal advice/ counselling**	**intensive help/ immediate intervention required**	**information/ personal advice/ counselling**	**intensive help/ immediate intervention required**	**parents vs professional**
Infancy review	15.3%	5.5%	0.9%	7.0%	0.6%	0.07
Somatic health	5.4%	11.4%	0.8%	17.9%	0.9%	<0.001
Motor development	1.0%	11.8%	0.4%	23.2%	0.3%	<0.001
Language, speech and cognitive development	0.8%	20.9%	0.2%	39.7%	0.2%	<0.001
Language use of parents	1.7%	11.1%	0.3%	23.9%	0.3%	<0.001
Emotional development	2.5%	22.4%	0.2%	38.6%	0.3%	<0.001
Contact between child and others	0.7%	8.9%	0.2%	16.7%	0.1%	<0.001
Child behavior	5.0%	27.7%	0.3%	47.7%	0.3%	<0.001
Parenting approach	2.9%	22.0%	0.4%	37.4%	0.6%	<0.001
Developmental stimulation	0.4%	11.6%	0.2%	27.1%	0.1%	<0.001
Time spending	0.7%	6.3%	0.5%	13.3%	0.4%	<0.001
Living environment	3.4%	3.0%	0.9%	7.2%	0.7%	<0.001
Social contacts	1.2%	3.1%	0.2%	5.1%	0.5%	<0.001
Day care for child	1.2%	2.0%	0.1%	4.4%	0.3%	<0.001
Concerns communicated by others	1.3%	2.4%	0.3%	5.1%	0.3%	<0.001
Family issues	8.8%	7.7%	1.7%	14.1%	2.3%	<0.001
Was any topic forgotten?	2.5%	15.7%	0.2%	18.7%	0.4%	<0.001

*The 6-point assessments of parents and professional were dichotomized for readability; category ‘no help needed’ was omitted. The comparison using Wilcoxon signed ranks test was on the full 6-point scale.

### Reliability

Concerning inter-rater reliability, ICCs were very high for physical topics (>0.85 to 1.0; see Table 3). For social-emotional topics, ICCs varied between 0.61 and 0.8. The ICC of the overall risk assessment was also very high: 0.92.

**Table 3:** Intra-class correlations for the interrater reliability of SPARK-domains

**Domain**	**ICC**
infancy review	0,953
somatic health	0,834
motor development	0,929
language-, speech- and cognitive development	0,877
language use of parents (second language, mother tongue)	0,801
emotional development	0,772
contact between child and others(both children and adults)	0,735
child behavior	0,899
parenting approach	0,618
developmental stimulation and early/preschool education	0,922
how the child spends its time	0,943
living environment in and outside the home	0,931
social contacts and informal support	0,908
day-care for the child	1,000
concerns communicated by others	0,763
family issues	0,857
overall risk assessment	0,925

### Validity

Convergent validity was low, with no correlations exceeding 0.3. Despite the low correlations, the pattern was as expected: higher scores (in this case above 0.1) were only found in domains that were expected to have higher correlations. Correlations above 0.2 include SPARK motor development with ASQ gross motor; SPARK language-, speech- and cognitive development with ASQ communication; SPARK child behavior with KIPPPI total score; SPARK family issues with KIPPPI life events (see Table 4). Domains of the NOSIK were not related to physically oriented SPARK domains, and significantly correlated to psychosocial domains. All correlations above 0.1 were significant at the 0.01 level.

**Table 4:** Convergent validity: correlations between perceived need for support on SPARK-domains and domain scores on self-report questionnaires

**perceived need for support (from CHC nurse)**	**Spear-man’s rho**	**ASQ communication**	**>ASQ gross motor**	**ASQ fine motor**	**ASQ problem solving**	**ASQ personal social**	**ASQ general**	**ASQ:SE total**	**ASQ:SE general**	**KIPPPI total score**	**KIPPPI Life Events**	**NOSIK**
infancy review	Corr.	-,047	-,037	-,073	-,049	-,024	,083	,069	-,095	,108	,087	,066
	Sig	,110	,212	,013	,101	,412	,005	,019	,001	,000	,004	,027
somatic health	Corr.	-,045	-,079	,017	-,042	-,033	,100	,084	-,103	,043	,006	,029
	Sig	,129	,008	,569	,159	,267	,001	,005	,000	,148	,844	,329
motor development	Corr.	-,104	-,224	-,075	-,053	-,060	,135	,051	-,052	,057	,056	,022
	Sig	,000	,000	,011	,076	,045	,000	,087	,081	,057	,061	,465
language-, speech- and cognitive development	Corr.	-,305	-,036	-,125	-,093	-,022	,124	,071	,027	,128	,007	,045
	Sig	,000	,226	,000	,002	,467	,000	,017	,361	,000	,810	,128
language use of parents	Corr.	,102	,063	-,076	-,072	-,015	,003	,150	-,038	,044	,104	,000
	Sig	,093	,298	,210	,249	,807	,956	,013	,532	,479	,096	,990
emotional development	Corr.	-,028	-,036	-,088	-,030	,025	-,019	,086	-,060	,141	,045	,168
	Sig	,352	,226	,003	,315	,410	,527	,004	,045	,000	,135	,000
contact between child and others	Corr.	-,031	-,004	-,019	-,091	-,019	,010	,093	-,042	,127	,052	,112
	Sig	,290	,891	,524	,002	,529	,736	,002	,156	,000	,086	,000
child behavior	Corr.	,024	-,012	-,042	-,062	-,030	,002	,148	-,159	,210	,046	,149
	Sig	,423	,684	,156	,038	,319	,945	,000	,000	,000	,123	,000
parenting approach	Corr.	-,062	-,023	-,049	-,069	-,002	-,003	,139	-,060	,167	,068	,156
	Sig	,037	,429	,098	,022	,950	,929	,000	,043	,000	,025	,000
developmental stimulation	Corr.	-,084	-,058	-,069	-,061	-,003	,043	,097	-,051	,098	,011	,018
	Sig	,005	,051	,020	,042	,910	,150	,001	,086	,001	,722	,555
how the child spends its time	Corr.	-,036	-,029	-,080	-,072	-,041	,012	,074	-,077	,096	,023	,108
	Sig	,230	,327	,007	,017	,176	,677	,013	,010	,001	,441	,000
living environment in and outside the home	Corr.	-,034	-,027	-,070	-,069	-,025	,016	,052	-,061	,050	,104	,044
	Sig	,257	,368	,019	,022	,409	,586	,079	,042	,099	,001	,143
(social contacts and informal support	Corr.	-,048	-,024	-,073	-,033	,000	,043	,042	,013	,069	,130	,081
	Sig	,105	,423	,014	,275	,992	,149	,160	,664	,022	,000	,006
day-care for the child	Corr.	-,031	-,018	-,053	-,025	,005	,046	,017	-,053	,061	,075	,063
	Sig	,304	,551	,075	,405	,855	,121	,575	,076	,044	,014	,036
concerns communicated by others	Corr.	-,072	-,046	-,047	-,049	-,048	,046	,064	-,105	,041	,022	,025
	Sig	,016	,121	,118	,101	,108	,119	,033	,000	,168	,465	,399
family issues	Corr.	-,041	,009	-,021	,001	-,017	,042	,084	-,060	,048	,230	,140
	Sig	,163	,760	,490	,967	,575	,162	,004	,045	,112	,000	,000
Something forgotten	Corr.	,124	,026	-,007	,040	,163	,022	,045	-,111	-,019	,035	,061
	Sig	,024	,642	,903	,475	,003	,688	,418	,045	,735	,527	,267

Corr = Spearman’s correlation. Sig = significance. Negative correlations are caused by differing scoring directions.

Analysis of groups based on SES-level showed that there was a highly significant difference in overall risk assessment (p < 0.001): there were relatively more children labeled as high risk in the lower SES groups compared to the groups with higher SES. There was also a small but significant difference in the level of parents’ concerns between SES-levels (median value range: 1.29 to 1.67, p < 0.001), but not in the perceived need for support (parents: 1.07 to 1.16; nurses: 1.19 to 1.30). The extreme-groups comparison followed almost the same pattern: significant differences in overall risk assessment (p < 0.001) and parental concerns (median value ‘reported’: 1.93 versus ‘everything OK’: 1.32, p = 0.043). There was a discrepancy in the perceived need for support: the reported children’s parents did not differ from the ‘everything OK’ children’s parents (1.13 vs 1.07, p = 0.60), but the need for support as perceived by the CHC-nurse was far higher for the reported children’s’ group (1.60 vs 1.19, p = 0.006). Table 5 shows the professional judgement of perceived need for support per domain, separately for the extreme groups and for the different SES-levels. The judgment was dichotomized for better readability into mild support (percentage *information wanted / personal advice / counselling*) and intensive support (percentage *intensive help/ immediate intervention required*). The reported group differed from the ‘everything OK’ group mostly in the domains related to the parent and family (parenting approach, living environment, social contacts, day care for child, concerns communicated by others, family issues, was any topic forgotten?). Lower SES-groups differed in a similar way from the higher SES-groups.

**Table 5:** Perceived need of support (professional assessment*) for extreme groups and SES-levels

% mild support /% intensive support *	**‘Everything OK’ group**	**Reported group**	**SES: very low N = 46**	**SES: low-avrg N = 433**	**SES: average N = 1237**	**SES: avrg-high N = 83**	**SES: high N = 22**	**SES: very high N = 38**
Infancy review	10.0% / -	7.7% / -	- / -	6.7% / 0.8%	7.1% / 0.6%	4.9% / 1.2%	13.6% / -	10.5% / -
Somatic health	24.6% / 3.3%	7.7% / 15.4%	19.0% / -	18.2% / 0.7%	18.0% / 1.1%	18.6% / 1.2%	9.0% / -	7.9% / -
Motor development	28.3% / -	30.8% / -	22.0% / -	22.6% / -	23.4% / 0.4%	22.5% / -	27.3% / -	15.8% / 2.6%
Language-, speech- and cognitive development	36.7% / -	23.3% / 7.7%	38.1% / -	39.3% / -	40.9% / 0.4%	38.3% / -	31.8% / -	52.6% / -
Language use of parents	14.3% / -	25.0% / -	28.6% / -	37.0% / -	21.1% / 0.4%	31.2% / -	- / -	16.7% / -
Emotional development	49.2% / -	46.2% / -	40.5% / -	39.7% / -	39.6% / 0.5%	32.9% / -	54.5% / -	18.4% / -
Contact between child and others	22.0% / -	7.7% / -	21.4% / -	22.1% / -	15.5% / 0.1%	46.9% / -	22.7% / -	10.5% / -
Child behavior	52.5% / -	38.5% / -	61.9% / -	46.6% / 0.5%	48.0% / 0.5%	46.9% / -	40.9% / -	39.5% / -
Parenting approach	34.4% / -	61.5% / -	64.3% / -	35.5% / 0.5%	37.4% / 0.8%	21.0% / -	45.5% / -	28.9% / -
Developmental stimulation	23.7% / -	30.8% / -	31.0% / -	28.1% / -	27.9% / 0.1%	13.6% / -	18.2% / -	18.4% / -
Time spending	10.3% / -	16.7% / -	21.4% / -	16.6% / 0.3%	11.8% / 0.4%	13.6% / -	13.3% / -	13.2% / -
Living environment	6.7% / -	36.4% / -	2.4% / -	11.2% / 0.5%	6.7% / 0.7%	4.9% / -	4.5% / -	5.4% / 2.6%
Social contacts	3.2% / -	- / 8.3%	2.4% / -	7.2% / 0.3%	5.5% / 0.5%	4.9% / -	- / -	- / -
Day care for child	6.6% / -	16.8% / 8.3%	9.5% / -	5.5% / -	5.9% / 0.4%	2.5% / -	4.5% / -	5.3% / -
Concerns commu-nicated by others	6.9% / -	33.4% / 8.3%	7.1% / -	5.3% / 0.3%	5.8% / 0.4%	3.7% / -	- / -	2.6% / -
Family issues	4.9% / -	58.3% / 25.0%	9.2% / 2.4%	17.6% / 2.8%	14% / 2.5%	8.6% / -	13.6% / -	10.5% / -
Was any topic forgotten?	22.2% / -	- / 20.0%	- / -	25.9% / -	18.0% / 1.0%	10.0% / -	22.2% / -	3.3% / -

*values indicate percentage mild support (*information wanted / personal advice / counselling*) and percentage intensive support (*intensive help/ immediate intervention required*); category ‘*no help needed*’ was omitted. SES = socio-economic status; SES-category ‘low’ had no cases. Avrg = average.

Furthermore, we found a difference in overall risk between children with and without completed self-report questionnaires. The group with completed questionnaires formed 66.9% of the total group, but included only 34.8% of the high risk labels. The group without questionnaires thus formed 33.1% of the total group, with 65.2% of the high risk labels. This difference in distribution is highly significant (p < 0.001).

### User experience

The survey on user experience was completed for a total of 211 contacts. Parents reported on 100 contacts, CHC-professionals on 179 contacts. After removing incomplete surveys, 86 parent-completed and 177 CHC nurse-completed surveys remained. Completing the survey took parents on average 5.2 minutes, and nurses 7.5 minutes. Both parents and CHC-nurses were positive about using the SPARK (satisfied or very satisfied about the contact: parents 94.2%; nurses 91.5%). Nurses succeeded in using the structured approach of the SPARK reasonably well to very well in 92.1% of the contacts. Despite the fact that the SPARK structured the visit, most parents and CHC-nurses found the visit very relaxed (89.6% and 65.6%). More than half of the parents regarded the information given during the visit as useful (66.3%) and tailored to their needs (58.1%). The majority of the parents (95%) reported that all relevant topics had been sufficiently discussed. CHC-nurses reported that using the SPARK provided them with information they would not have collected without using such a structured instrument, especially regarding topics related to family matters (25.4% of the contacts), parenting approach (15.8%) and concerns communicated by others (11.9%). The results of the survey were discussed with the same expert group of CHC nurses that had helped develop the SPARK (n = 8) [15]. The results of the survey and this discussion resulted in the following comments on using and improving the SPARK. The SPARK supports the CHC-nurse in making difficult visits: it ensures that nothing is forgotten, and helps in asking tough questions. Asking for the concerns and needs of parents gives much additional information in families with problems, which helps in deciding what care should be offered to these families. However, in families where everything is OK, the SPARK was found to be too rigid. Furthermore, the expert group reported that the wording of the answering categories of the question whether parents experienced had any concerns, questions or problems in the last six months needed improvement.

## Discussion

This study assesses the psychometric properties of the SPARK, a structured interview developed to assess parenting and developmental problems in young children. The inter-rater reliability was found to be very good to excellent, especially for the overall risk assessment and the physical domains. The SPARK showed to be discriminative, by distinguishing between areas with different SES-levels and between postal codes (representing both SES and urbanization). There were clear differences between extreme groups: children reported to the child protective services versus children with positive scores only on all questionnaires. The only psychometric property that was below expectation was the convergent validity. Correlations of SPARK-domains with related domains in the self-reported questionnaires were significant, but very low. Although they showed the expected pattern, no correlation exceeded 0.3. This lack of convergence is probably influenced by several aspects. Firstly, the content and the way of questioning differed quite a lot between the SPARK and the self-report questionnaires. Secondly, the majority of the children had no problems. Thirdly, the group that did not return the questionnaires included a large part of the children with a high risk. Both parents and CHC-nurses were positive about the SPARK. CHC-nurses reported that the SPARK gave practical information and supported them during visits with problem families. They also identified several areas of improvement for the SPARK: its rigid structure and the wording of some questions.

Several authors support our opinion that an assessment of parents’ concerns and their need for support should be done in dialogue with the parents [32-34]. One of the main features of the SPARK is direct interaction between parent and professional: the focus is on interactively discussing with parents the child’s needs and development and their needs for parenting support. This professional helps the parent with arranging and judging concerns and problems. The only instrument that has a somewhat similar approach to the SPARK is the Parents’ Evaluations of Developmental Status (PEDS) by Glascoe [33,35]. However, there are some major differences between the PEDS and the SPARK. The PEDS is a short 10-item questionnaire to be completed before a visit to a pediatric clinic using a self-report or interview [33,35]. The answers are then discussed by the nurse or pediatrician. The SPARK differs from the PEDS in that it is a conversation between parent and professional in order to clarify care needs and to jointly decide on subsequent care. Both the parents and the professional rate their perceived need for support, which is important in situations when parents are avoiding care and to reveal differences in the perceived need between parents and professional. Furthermore, the SPARK has a broader scope, including also the child’s environment. Finally, the SPARK results in an overall assessment of risk for parenting and developmental problems. Whether the SPARK is preferable to self-report questionnaires needs to be determined. The duration of administering the SPARK is about double that of the regular time spent in a visit to the well-baby clinic. This will hamper implementation, in the Netherlands as well as in other countries. Further research is needed on whether implementing the SPARK is cost-effective. Three arguments are in favor of the SPARK: *a)* in our current study we observe a response bias, as especially the parents with a child labeled as high risk by the nurse did not return the self-report questionnaires, *b)* the interview gives nurses the possibility to ask not only about the child, but also about the (functioning of) the family. Nurses reported that this part in particular gave them new information relevant for deciding which care and support should be offered, and *c)* in the Netherlands there is a growing aversion among parents to self-report questionnaires. Parents regard preventive child health care increasingly as a system for detection of child abuse and neglect, instead of as a care provider that supports parents of young children [36]. This threatens the high reach (>95%) that the Dutch system has traditionally had between 0–4 years. The interactive procedure of the SPARK (i.e. listening to the parent and making a shared decision about subsequent care) may help in re-establishing the trust of parents in preventive child health care.

This study has several limitations. The low convergent validity needs further attention. In addition to the reasons stated above, some other aspects play a role. Firstly, although the response rate for the self-report questionnaires was quite high, there was selective non-reporting: about two-thirds of the children with a label of high risk were part of the one-third that did not return questionnaires. This may have negatively influenced the convergent validity, as the group with expected high scores in both the SPARK and the self-report questionnaires did not contribute to the correlations. Interestingly, this lower response rate showed that the SPARK identifies a large group of children with high risk for parenting problems, which would have been missed by using only self-report questionnaires. Reasons for not returning the questionnaires are unknown, but may include causes as diverse as lack of skills to complete a self-report questionnaire, stress within the family, or not wanting to write about problems within the family. Secondly, we were limited in choosing suitable questionnaires as there is a lack of validated questionnaires for this age group in the Dutch language. Some of the instruments used for assessing the convergent validity have been validated only partially (the KIPPPI, which is used extensively in the Netherlands) or have not been validated for this age group in the Netherlands (ASQ and ASQ:SE). This limits the interpretability of the convergent validity. Thirdly, the lack of convergence may also have been caused by the broad scope of the SPARK compared to the more limited self-report questionnaires.

Another limitation is that, although the province of Zeeland resembles a large part of the Netherlands, it may not be representative of some highly urbanized areas elsewhere in the Netherlands. The validity and feasibility of the SPARK in urbanized, multi-ethnic areas should also be studied. Also, this was a cross-sectional study without follow-up. Further study is required to assess the predictive validity of the SPARK and long-term outcomes.

## Conclusion

The SPARK is a structured interview that assesses parents’ concerns and their need for support using both the parents’ perspective and the experience of the CHC-nurse. The SPARK discriminates between children with a high, increased and low risk for parenting and developmental problems in a reliable way. The SPARK is practicable and provides useful information which helps to decide, together with the parents, what care is needed in a family. The users are satisfied, but there is room for improving the instrument. Several aspects of the SPARK such as predictive validity, construct validity, cost-effectiveness and discriminative validity in other samples require further study. By using only self-report questionnaires, a large part of the children with a high risk on parenting and developmental problems is left out.

## Abbreviations

ASQ: Ages and stages questionnaire; ASQ:SE: Ages and stages questionnaire: social emotional; CHC: Child health care; ICC: Intraclass correlation; KIPPPI: Kort Instrument voor de Psychologische en Pedagogische Probleem Inventarisatie; NOSIK: Nijmeegse ouderlijke stress index – kort; PEDS: Parents’ evaluations of developmental status; SES: Socio-economic status; SPARK: Structured problem analysis of raising kids.

## Competing interests

The authors declare that they have no competing interests.

## Authors’ contributions

HFvS has conceived the study, participated in its design and coordination, performed the statistical analysis and drafted the manuscript. IEES has conceived the study, participated in its design and coordination, collected the data, participated in the statistical analysis and helped to draft the manuscript. JMAH and AJPS have participated in the design of the study, and revised the manuscript critically for important intellectual content. All authors have read and approved the final manuscript.

## Pre-publication history

The pre-publication history for this paper can be accessed here:

http://www.biomedcentral.com/1471-2431/12/71/prepub

## References

[B1] Bricker D Davis Schoen M Squires J Mental health screening in young children Infants & Young Children 2004 17 129 144 10.1097/00001163-200404000-00005

[B2] Hertzman C Siddiqi A Hertzman E Irwin LG Vaghri Z Houweling TAJ Tackling inequality: get them while they're young BMJ 2010 340 346 348 10.1136/bmj.c346 20147358

[B3] van Leerdam F Raat H Hira Sing R Update programmeringsstudie effectonderzoek JGZ 0–19 jaar, evidence in de literatuur. Update research programming for youth health care 0–19 years, evidence in literature Tijdschrift voor Jeugdgezondheidszorg 2006 38 38 57

[B4] Moran P Ghate D van der Merwe A What Works in Parenting Support? A Review of the International Evidence. RR574 2004 DfES Publications, Nottingham

[B5] Chief Secretary to the Treasury Every Child Matters 2003 The Stationery Office, Norwich

[B6] Hermanns J Ory F Schrijvers AJP Helpen bij opgroeien en opvoeden: eerder, sneller en beter. [Supporting development and parenting: sooner, faster, and better: an advice for early detection and interventions in regard to developmental and parenting problems.] 2005 Invent groep, Utrecht

[B7] Glascoe FP Screening for developmental and behavioral problems Ment Retard Dev Disabil Res Rev 2005 11 173 179 10.1002/mrdd.20068 16161092

[B8] Bouwmeester-Landweer M Early Home Visitation in Families at Risk for Child Maltreatment 2006 Optima Grafische Communicatie, Rotterdam

[B9] Junger M De oorsprong van geweld. [The origin of violence.] 2004 Vossiuspers UvA, Amsterdam

[B10] Tremblay RE Nagin DS Séquin JR Zoccolillo M Zelazo PD Boivin M Physical agression during early childhood: trajectories and predictors Pediatrics 2004 114 43 50 10.1542/peds.114.1.e43 PMC3283570 15231972

[B11] Carneiro PM Heckman JJ Human Capital Policy IZA Discussion Paper No. 821. Available at SSRN: http://ssrn.com/abstract=434544

[B12] Barlow J Schrader McMillan A Kirkpatrick S Ghate D Smith M Barnes J Health-led Parenting Interventions in Pregnancy and Early Years. DCSF-RW070 2008 University of Warwick, Coventry

[B13] Vogels T Reijneveld SA Brugman E den Hollander-Gijsman M Verhulst FC Verloove-Vanhorick SP Detecting psychosocial problems among 5-6-year-old children in preventive Child Health Care: the validity of a short questionnaire used in an assessment procedure for detecting psychosocial problems among children Eur J Public Health 2003 13 353 360 10.1093/eurpub/13.4.353 14703324

[B14] Postma S Guideline early detection psychosocial problems in preventive child health care (in Dutch). 295001002 2008 RIVM, Bilthoven

[B15] Staal IIE van den Brink HAG Hermanns JMA Schrijvers AJP van Stel HF Assessment of parenting and developmental problems in toddlers: development and feasibility of a structured interview Child Care Health Dev 2011 37 503 511 10.1111/j.1365-2214.2011.01228.x 21434969

[B16] Kijlstra M Prinsen B Schulpen T Kwetsbaar jong! [Vulnerable young!] 2002 NIZW, Utrecht

[B17] Sidebotham P Heron J The ALSPAC Study Team University of Bristol The ALSPAC Study Team University of Bristol Child maltreatment in the "children of the nineties": A cohort study of risk factors Child Abuse Negl 2006 2005 30 497 522 16701895 10.1016/j.chiabu.2005.11.005

[B18] Squires J Bricker D Potter L Revision of a parent-completed development screening tool: Ages and Stages Questionnaires J Pediatr Psychol 1997 22 313 328 10.1093/jpepsy/22.3.313 9212550

[B19] Squires J Potter L Bricker DD The ASQ User's Guide for the Ages & Stages Questionnaires: a parent-completed, child-monitoring system 2003 2 Paul H. Brookes Publishing Co, Baltimore

[B20] Squires J Bricker DD Twombly E The ASQ:SE User's Guide for the Ages & Stages Questionnaires: Social-Emotional. A parent-completed, child-monitoring system for social-emotional behaviours 2003 Paul H. Brookes Publishing Co, Baltimore

[B21] Abidin RR Parenting stress and the utilization of pediatric services Child Health Care 1983 11 70 73 10262147 10.1207/s15326888chc1102_5

[B22] de Brock AJLL Gerris JRM Abidin RR [Manual NOSI] Handleiding NOSI 1992 Harcourt Test Publishers, Amsterdam

[B23] Romijn A Kousemaker NPJ [The KIPPPI-method for early detection. Revision and further justification] De KIPPPI-methode voor vroegtijdige onderkenning. Revisie en nadere verantwoording 2001 Faculteit Sociale Wetenschappen, Universiteit Leiden, Leiden

[B24] Janson H Squires J Parent-completed developmental screening in a Norwegian population sample: a comparison with US normative data Acta Paediatr 2004 93 1525 1529 10.1111/j.1651-2227.2004.tb02641.x 15513584

[B25] Kapci EG Kucuker S Uslu RI How applicable are ages and stages questionnaires for use with Turkish children? Topics in Early Childhood Special Education 2010 30 176 188 10.1177/0271121410373149

[B26] Kucuker S Kapci EG Uslu RI Evaluation of the Turkish Version of the Ages and Stages Questionnaires: Social-Emotional in Identifying Children With Social-Emotional Problems Infants & Young Children 2011 24 207 220 10.1097/IYC.0b013e31820eae26

[B27] Kerstjens JM Bos AF ten Vergert EMJ de Meer G Butcher PR Reijneveld SA Support for the global feasibility of the Ages and Stages Questionnaire as developmental screener Early Hum Dev 2009 85 7 443 447 10.1016/j.earlhumdev.2009.03.001 19356866

[B28] Caris CJ [Lets talk. A study on parenting support at the well-baby clinic]. Laten praten. Een onderzoek naar opvoedingsondersteuning op het consultatiebureau 1997 SWP uitgeverij, Utrecht

[B29] Streiner DL Norman GR Health measurement scales. A practical guide to their development and use 2003 3 Oxford University Press, Oxford

[B30] Campbell DT Fiske DW Convergent and discriminant validation by the multitrait-multimethod matrix Psychol Bull 1959 56 81 105 13634291

[B31] Altman DG Practical Statistics for Medical Research 1991 Chapman & Hall, London

[B32] Glascoe FP Marks KP Detecting children with developmental behavioral problems: The value of collaborating with parents Psychological Test and Assessment Modeling 2011 53 258 279

[B33] Glascoe FP Evidence-based approach to developmental and behavioural surveillance using parents' concerns Child Care Health Dev 2000 26 137 149 10.1046/j.1365-2214.2000.00173.x 10759753

[B34] Puura K Davis H Papadopoulou K Tsiantis J Ispanovic-Radojkovic V Rudic N The European Early Promotion Project: A new primary health care service to promote children's mental health Infant Ment Health J 2002 23 606 624 10.1002/imhj.10039

[B35] Glascoe FP Early detection of developmental and behavioral problems Pediatr Rev 2000 21 272 279 10.1542/pir.21-8-272 10922024

[B36] Pardoen J Boeke H [Code Orange (be alert). The The fragile confidence of parents in preventive child health care] Code Oranje (wees alert). Het kwetsbare vertrouwen van ouders in de jeugdgezondheidszorg 2011 Ouders online, Amsterdam

